# Vascular Tension Gone Wrong: Sigmoid Colon Herniation Leading to a Mesenteric Hematoma

**DOI:** 10.7759/cureus.78786

**Published:** 2025-02-09

**Authors:** Daniel Hahn, Lauren Velasquez, Marc R Mohammed, Albert Cooper

**Affiliations:** 1 Medicine and Surgery, Touro College of Osteopathic Medicine, New York, USA; 2 Surgery, One Brooklyn Health, Interfaith Medical Center, Brooklyn, USA

**Keywords:** inguinal hernia, mesenteric hematoma, sigmoid colon hernia, traction injury, vascular injury

## Abstract

Mesenteric hematomas, a rare and clinically significant condition, are typically associated with abdominal trauma, vascular conditions, or surgical complications. Spontaneous mesenteric hematomas, an even rarer subtype, are often seen in patients who are on anticoagulation therapy or have inguinal hernias. In this case report, we discuss an 89-year-old male patient who initially presented with stable vital signs and abdominal pain but was later discovered to have a large, stable mesenteric hematoma on an abdominal computed tomography scan. The hematoma was believed to be secondary to the herniated sigmoid colon exerting tension on the mesenteric vasculature. This case represented an unusual interaction between sigmoid colon hernias and spontaneous mesenteric hematoma formations, underscoring the importance of considering mesenteric vascular injury in patients with non-surgically managed hernias.

## Introduction

Mesenteric hematomas are a rare but clinically significant condition that is often associated with trauma, vascular disease, aneurysms, or perioperative complications [1]. A spontaneous mesenteric hematoma is an even rarer presentation that is frequently linked with anticoagulant use, inflammatory gastrointestinal diseases such as pancreatitis, connective disease disorders, malignancy, or unknown etiology [1-3]. The presentation of a spontaneous mesenteric hematoma can be highly variable-from asymptomatic to acute pain and shock-based on the location, size, hemodynamic stability, and associated vascular supply to the hematoma [4,5]. This diagnosis may be challenging, requiring prompt, repeated imaging with abdominal computed tomography (CT) scans and CT angiography [4].

Spontaneous mesenteric hematomas, a significant and life-threatening complication, are not a commonly discussed complication of hernias that are managed non-surgically. The present case describes a stable, spontaneous hematoma as a complication of an inguinal herniation of the sigmoid colon. Furthermore, there was an unusual occurrence where the spontaneous mesenteric hematoma obstructed the intraoperative reduction of the sigmoid colon. This case underscores the complex interplay between a hernia, mesenteric vascular injury, and the risk of potential incarceration.

## Case presentation

The patient, an 89-year-old man with a complex medical history including hypertension, diabetes mellitus, right-sided hydrocele, cerebrovascular accident with right-sided weakness, abdominal aortic aneurysm, and a left-sided reducible inguinal hernia being medically managed, presented to the emergency room with complaints of urinary incontinence, urinary retention, and abdominal pain. The patient had reported normal bowel movements and denied nausea, vomiting, melena, and hematochezia. On physical exam, the patient had a swollen scrotum consistent with a previously known right-sided hydrocele. The scrotum had no associated erythema or warmth. Palpation of the abdomen was soft, with voluntary guarding on deep palpation and no palpable mass. The patient reported that he was not currently taking anticoagulants. Initial laboratory studies showed a red blood cell (RBC) count of 3.49 x 10^6^/μL, hemoglobin of 10.9 g/dL, and hematocrit of 33.2%.

Imaging

**Figure 1:**
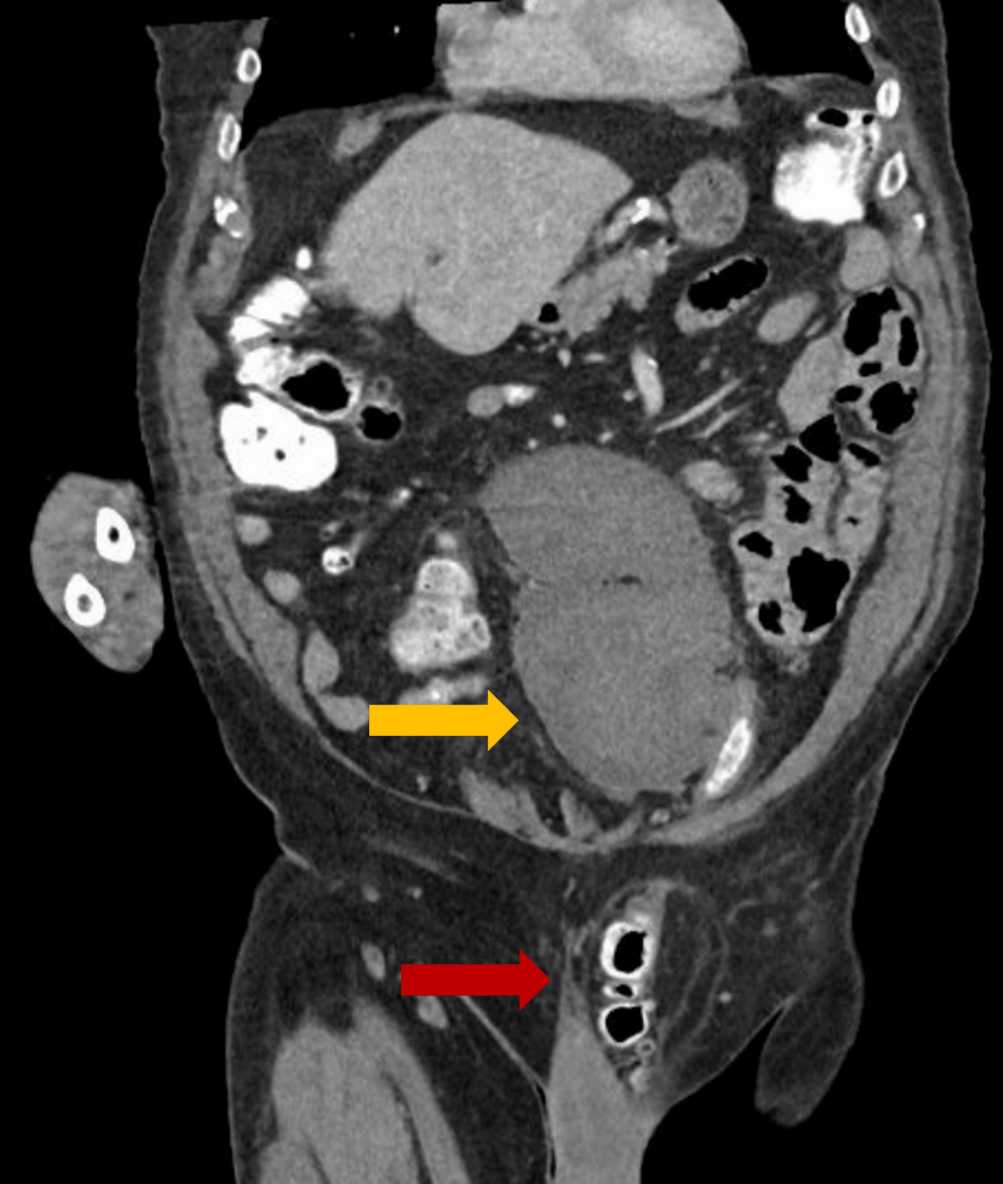
Abdominal Computed Tomography Scan of the Sigmoid Colon Hernia and Mesenteric Hematoma Orange arrow: mesenteric hematoma; red arrow: sigmoid colon hernia

Abdominal CT showed a mesenteric hematoma of the midabdominal mesentery extending to the left anterior pelvis with a bilobed appearance measuring up to 14 cm in the superior-inferior axis (Figure 1). The sigmoid colon was mildly thickened and decompressed. The distal large bowel protruded into the left inguinal hernia sac, measuring 13.0 cm long, and extended nearly to the scrotum, measuring 5.7 cm. No small or large bowel obstruction thickening was identified.

**Figure 2:**
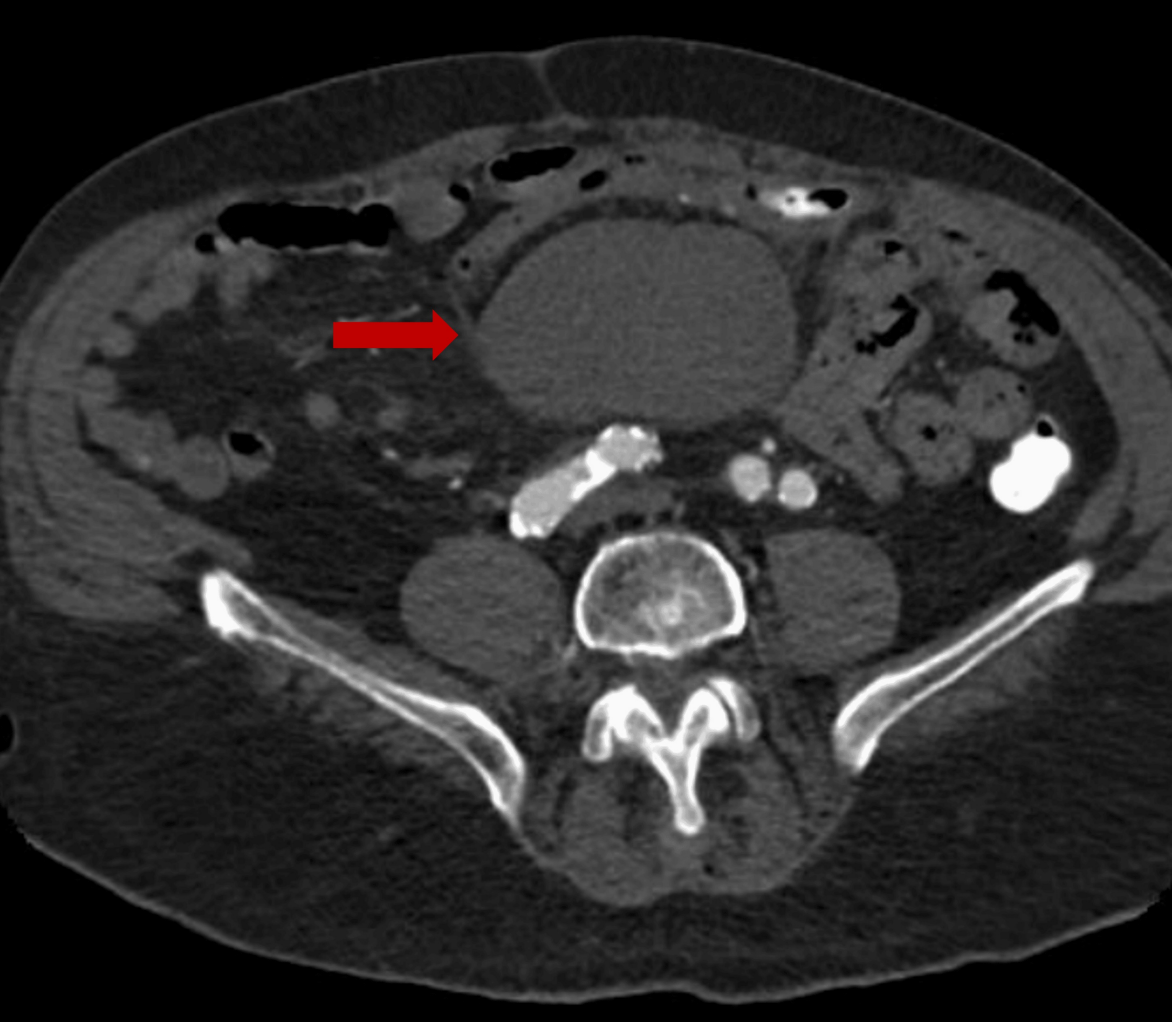
Abdominal Computed Tomography Scan of the Mesenteric Hematoma Red arrow: mesenteric hematoma

The upper portion transversely measured at 8.0 x 5.0 cm, and the lower portion measured at 6.2 x 9.4 cm (Figure 2). The hematoma was located directly adjacent to the distal sigmoid colon. Imaging showed no evidence of internal active bleeding or accumulation of contrast material.

Operation report

The surgeon performed an exploratory laparotomy to repair the incarcerated left inguinal with mesh and to evacuate the sizeable mesenteric hematoma, in order to alleviate the patient's abdominal discomfort. An inguinal incision was made through Camper’s fascia, Scarpa’s fascia, and the aponeurosis of the external oblique. The fascia was opened, and the ilioinguinal nerve was identified and preserved. The large hernia sac was identified anteromedial to the spermatic cord. The hernia sac was opened. It contained a hematoma and an incarcerated sigmoid colon. The hematoma was aspirated. Careful adhesion lysis was carried out to free the colon from the hernia sac.

Due to the inability to reduce the sigmoid colon from the inguinal incision, a lower midline abdominal incision was made and taken down to the fascia. It was discovered that there was a sizeable mesenteric hematoma pushing against the deep inguinal ring, preventing the reduction of the sigmoid colon. Therefore, the chronic hematoma had to be first decompressed through its wall with a purse string incision. The hematoma contained old, clotted blood, but there was no active bleeding. Once the hematoma was decompressed, the sigmoid colon was reduced into the abdominal cavity. A large mesh was used to plug the hernia sac, which then was attached to the adjacent tendon. A Jackson-Pratt drain was left in the peritoneal cavity near the hematoma to monitor a potential post-operative reappearance of the hematoma. The patient tolerated the procedure well, the mesenteric hematoma did not reappear, and the patient was discharged to the intensive care unit.

## Discussion

Inguinal hernias are a relatively common clinical condition that affects approximately 6% of elderly patients. Of these cases, approximately 10% are associated with incarcerations, strangulations, or intestinal obstructions [6]. Within inguinal hernia sacs, different anatomical contents may be found: in left-sided hernias, the sigmoid colon is a structure that is often seen, especially with sliding hernias. As the sigmoid colon enters the hernia sac, it exerts tension on the mesentery, which can cause an injury, such as a mesenteric hematoma. The present case describes a unique clinical picture with only one similar report in the literature. Sutton et al. reported a case of a 67-year-old man with a spontaneous mesenteric hematoma caused by a recurrent inguinal hernia that became incarcerated and produced a traction injury to the mesentery [7].

A mesenteric hematoma is a rare condition classically precipitated by traumatic causes or mesenteric vascular disease [1]. Spontaneous mesenteric hematomas not caused by the previously described clinical features are typically due to anticoagulant use, collagen diseases, or other gastrointestinal diseases such as Crohn’s disease [3]. The presenting symptoms are non-specific, including abdominal pain, abdominal masses, nausea, vomiting, diarrhea, and melena. An acute hematoma may also present with hypotension. This hematoma’s stable nature at presentation; the presence of old, clotted blood; a normotensive status; and gradual onset suggest that the patient’s bleeding originated from a mesenteric vein rather than an arterial source.

Conservative management, such as hemodynamic monitoring, correction of coagulopathy, and evaluation of possible complications, is often adequate for small or stable hematomas [4]. However, this hematoma’s significant size, volume, and location adjacent to the sigmoid colon added further management complexities. Surgical intervention in large hematomas could lead to rapid loss of tone and cardiovascular collapse [4]. Therefore, careful consideration should be given to maintaining hemostasis.

In this case, the hematoma directly obstructed surgical attempts to reduce the incarcerated hernia. If a hematoma can affect the hernia’s reduction, additional preliminary diagnostic imaging and/or surgical interventions, such as a hematoma evacuation or decompression, should be considered before addressing the hernia. In a cyclic relationship, inguinal hernia repairs have also been shown to be associated with bleeding complications, including the formation of hematomas [8]. This case presentation highlights the importance of monitoring post-operative hematoma formations because of their potential to complicate reoperative procedures.

## Conclusions

This case highlights the unusual presentation of having both a sigmoid colon hernia and a large spontaneous mesenteric hematoma that obstructed the reduction of the incarcerated inguinal hernia. This clinical presentation underscores the importance of having a high index of suspicion for hematoma formation, especially in patients with long-standing hernias who are not on anticoagulant therapy. Concurrent conditions require physicians to have flexible surgical strategies and a low threshold for repeat diagnostic imaging. This report adds to the limited literature on traction-related mesenteric injury and establishes a bidirectional hematoma-hernia interrelationship.
